# Medical professionals’ job satisfaction and telemedicine readiness during the COVID-19 pandemic: solutions to improve medical practice in Egypt

**DOI:** 10.1186/s42506-023-00127-7

**Published:** 2023-03-07

**Authors:** Hanan El-Mazahy, Jaidaa Mekky, Noha Elshaer

**Affiliations:** 1 Mental Health, Private Practice, Alexandria, Egypt; 2 Department of Neuropsychiatry, Faculty of Medicine, Alexandria University, Alexandria, Egypt; 3 Industrial Medicine and Occupational Health, Department of Community Medicine, Faculty of Medicine, Alexandria University, Alexandria, Egypt

**Keywords:** COVID-19, Job satisfaction, Medical professionals, Telemedicine

## Abstract

**Background:**

The COVID-19 pandemic has impacted medical professionals’ job satisfaction and was a call to adopt telemedicine. Finding out how far medical professionals are satisfied and ready to use telemedicine would be important to improve medical practice.

**Methods:**

Data was collected from 959 medical professionals from both the governmental and private health sectors in Egypt in 2021 using a specifically designed online questionnaire, to evaluate job satisfaction, perception of telemedicine, and propose solutions to improve medical practice.

**Results:**

The study revealed low to moderate job satisfaction at governmental (27.2%) and private (58.7%) sectors. Underpayment was the most reported challenge at both sectors (37.8% and 28.3%, respectively). Dissatisfaction with government salary was independently predicted by working at the Ministry of Health and Population (OR = 5.54, 95%CI = 2.39,12.8; *p* < 0.001). Wage increase (46.10%), medical training of professionals (18.1%), and management of non-human resources (14.4%) were the most proposed solutions to improve medical practice in Egypt. During the COVID-19 pandemic, 90.7% of medical professionals had practiced telemedicine with moderate level of perception of its benefits (56%).

**Conclusions:**

During the COVID-19 pandemic, medical professionals reported low to moderate job satisfaction and a moderate level of perception of telemedicine. It is recommended to analyze the healthcare financing system and provide continuous training of medical professionals to improve medical practice in Egypt.

## Introduction

During the COVID-19 pandemic, a framework for telemedicine implementation was defined [1–3]. However, a systematic review in 2021 indicated that in some countries, certain gaps hindered its implementation such as the lack of rules and regulations regarding the utilization of telemedicine and a lack of telemedicine system integration and infrastructure [4]. In countries with a lack of regulations to authorize and integrate telemedicine services in their national healthcare system, the COVID-19 pandemic was a call to adopt regulations that support broad telemedicine implementation [1, 5–7].

Egypt has taken steady steps in the direction of digital transformation; this has been amplified with the COVID-19 crisis, where the Information and Communications Technology sector played an important role through the use of applications that enable organizations to continue their operations while considering health and safety measures [8]. However, implementation of telemedicine in Egypt, like other countries, relies on essential elements; namely, the technology in terms of its availability, training, usability, and quality aspects. In addition, acceptance, financing, organization, and policy and legislation ensuring standardization and security are all required [9, 10].

Technology acceptance of both patients and professionals has been identified as the second most reported determinant of a successful telemedicine implementation [9]. The national Egyptian survey conducted in 2020 evaluated the attitude towards telemedicine among the general population. Half of the participants stated that they had formerly used a telemedicine tool mainly video or phone call (39.3%) and mobile application (23.7%). Two thirds of the participants were more likely to favor telemedicine than conventional ways, yet 13.7% found it challenging. The survey results suggested that the majority of Egyptians appear to perceive the benefits of telemedicine positively [11].

On the contrary, finding out how far medical professionals are ready to accept and use telemedicine in Egypt has been evaluated in small studies in certain specialties [12, 13]. Studies conducted in other countries during the COVID-19 crisis to evaluate physicians’ perception of telemedicine revealed variable results; for example, a study conducted in northeast Florida in 2020 found physicians had a positive attitude towards the adoption of telemedicine and perceived the quality of health service delivery as comparable to in-person care [14]. On the contrary, in Ireland, health care providers did not universally accepted this mode of health care delivery [15]. A study in the USA 2021 suggested that implementation of telemedicine might be easiest among physicians who perceive the effectiveness of telemedicine, value technology-based workstyle, and have higher satisfaction [16].

High levels of job dissatisfaction were reported among physicians during the COVID-19 pandemic in Ghana and Kenya [17]. A study conducted in Jordan 2021 revealed that being a general practitioner or specialist, working at highly loaded hospitals, suffering from burnout, and low salaries have predicted lower levels of job satisfaction among health care providers during the COVID-19 pandemic [18]. It has been reported that the COVID-19 crisis had an impact on the physical and psychological wellbeing of health care providers in low-, middle-, and high-income countries. Work-related stress, insomnia, anxiety, and depression were prevalent among health care providers, leaving them vulnerable to suffer from burnout which could lead to a deterioration in the level of job satisfaction [17–19].

Egypt suffers from a shortage of physicians although an average of 10,000 medical students graduate annually [20]. The shortage is believed to be due to the emigration of both qualified trainers and graduates, which is attributed mainly to low job satisfaction [21, 22] and underpayment that has resulted in Egyptian junior doctors leaving their country, especially since some developed countries have easy licensing and registration requirements [20, 23]. A survey conducted in Egypt 2015 found that 85.7% of undergraduate medical students intended to leave Egypt after graduation [22]. Medical professionals’ job satisfaction is a cornerstone for improving the quality of health care [21]. Defining the challenges in medical practice and telemedicine implementation in Egypt is crucial to suggest solutions. The current study was conducted among medical professionals in Egypt during the COVID-19 pandemic to evaluate job satisfaction and perception of the benefits of telemedicine, identify challenges, and propose solutions to improve medical practice.

## Methods

### Study design, sample, and setting

A cross-sectional online survey was conducted between September and November 2021, among medical professionals in Egypt. The questionnaire was administered on particular sites on the Internet which are frequently and specifically visited by medical professionals in the country. The selected sample (*n* = 959) provided information representing male and female medical professionals at the governmental and/or private health sectors in Egypt. Most medical specialties were represented in this study. Using an online survey enabled reaching a large number of the target population in different geographic localities of the country at a fraction of the cost and time compared with a face-to-face survey.

### Participants

The target population were medical professionals in any health field currently working at the governmental and/or private health sectors in Egypt. A total of 971 medical professionals participated in this survey. The screening of the 971 responses resulted in exclusion of 12 participants; two retired, six working abroad at survey time, and four with incomplete questionnaires with more than 15% missing data. Responses of 959 participants have been included in the analysis.

### Research tool

Data were collected using a specifically designed questionnaire including 22 questions as follows:

- Section (I) included 9 questions on demographic data (gender, age, highest qualification attained, and medical specialty), health sector (governmental, private or dual governmental-private), and workplace within the governmental sector (University Teaching Hospital, Ministry of Health and Population (MoHP), Health Insurance Organization (HIO)).
- Section (II) included 4 close-ended questions to specify the level of satisfaction with the job, and salary regarding governmental or private work, coded on a 4-point Likert rating scale (1 = very dissatisfied, 2 = dissatisfied, 3 = satisfied, and 4 = very satisfied).
- Section (III) included five open-ended questions to indicate pros and cons of working at the governmental or private sector and suggested solutions to improve medical practice in Egypt, as perceived by the participants.
- Section (IV) included 4 questions about telemedicine practice during the COVID-19 pandemic. Participants were asked to report whether they use telecommunication infrastructure to deliver health consultations, and/or results of investigations (answered by yes or no), perception of the benefits of telemedicine (answered by not beneficial at all, beneficial, has some benefits and some limitations, or do not know). This was followed by two open-ended questions to state the perceived benefits and barriers of telemedicine.

### Data management (open-ended question analysis)

Open-ended questions (7 questions) allowed authentic feedback by giving the participants a chance to express their opinion in their own words. Responses were coded using the “inductive coding” method, where codes arose directly from the survey responses without any predefined set of codes. Though inductive coding is difficult, it was preferred because it is less prone to bias. This method demanded reading every response as follows: reading one response, creating a code to cover it, then reading another response, and applying a previous code or creating a new one to cover it. Throughout that process, a review of codes was done frequently to adjust the codes (split an existing code into two or change its description to cover more responses). More than one researcher interpreted every response, to ensure using multiple perspectives to interpret a single set of data.

Responses from the survey were exported to a Microsoft Excel file, where an individual response was recorded by placing “1” in each cell where an answer (in a row) matched a category code (in column) to indicate positive response in this category. Sometimes, a single answer entailed multiple different positive responses; this was recorded by placing “1” in every matched category code. Finally, the developed codes were organized into groups of codes related to one another, to create a “hierarchical coding frame” which was more powerful and allowed different levels of granularity during the analysis of the results. For instance, the solutions for the improvement of medical practice were organized into six categories according to the World Health Organization health systems’ framework [24].

### Reliability and validity of the research tool

Cronbach Alpha reliability [25] of the generated scale measuring satisfaction with job and salary at governmental work (2 items) and private work (2 items) was 0.96 and 0.97, respectively. In literature, it has been mentioned that for qualitative data (open-ended questions), reliability was found to be superfluous, whereas validity “objectivity” is centered on the interpreter (unlike quantitative research where the objectivity is centered on the instrumentation utilized) [19, 20]. Accordingly, in this study, the abovementioned data management steps were taken to avoid bias and ensure coverage, flexibility, accuracy, and validity [26, 27].

### Statistical analysis of the data

The coded data were transferred from Microsoft Excel to an SPSS file (SPSS v.22; IBM Corp. Released 2011. IBM SPSS Statistics for Mac, Armonk, NY, USA) for data analysis. Data were described using frequencies and percentages. A case–control approach analysis using univariate logistic regression was conducted to compute the odds ratio (OR) and associated 95% confidence interval (95% CI) to quantity the extent of satisfaction with job and salary associated with demographic characteristics and workplace. Since some participants had only one job in one sector, the aforementioned analysis was conducted independently at the governmental and private sector. Multivariate logistic regression analysis was conducted to model dissatisfaction with the government salary as a function of demographic factors and workplace in order to study their independent effect. The model included professionals at the governmental sector who reported a level of satisfaction with salary (*n* = 827). It included four factors namely gender, age, medical specialty, and workplace. The adequacy of the model in data fitting was determined by Nagelkerke’s *R*^2^ and Hosmer and Lemeshow goodness-of-fit test. All statistical analyses were judged at a 5% level of significance (*α* = 0.05).

## Results

### Sociodemographic characteristics and workplace

More than two thirds of the enrolled professionals were internists (66.1%), and 33.9% were surgeons. Just less than two thirds had dual government-private jobs (63.4%), whereas some had only one job at the governmental (27.2%) or private (9.4%) sector (Table 1).

**Table 1:** Sociodemographic characteristics and workplace of medical professionals in Egypt, 2021 (*n* = 959)

Characteristics	Frequency (no.)	Percentage (%)
Gender		
Male	576	60.1
Female	383	39.9
Age (years)		
23 ≤ 30	245	25.5
30 ≤ 40	552	57.6
40 ≤ 50	119	12.4
≥ 50	43	4.5
Highest qualification attained		
MBBCh (or BScD)	355	37.0
Postgraduate diploma	115	12.0
Master’s degree	357	37.2
Doctorate degree (or PhD)	61	6.4
Egyptian board certificate	51	5.3
Non-Egyptian board certificate	20	2.1
Medical specialty		
General and internal medicine^b^	634	66.1
Pediatrics and neonatology	159	25.0
General practitioner	61	9.6
Emergency medicine	48	7.6
Diagnostic radiology and imaging	45	7.1
Internal medicine (general)	42	6.6
Neuropsychiatry	41	6.5
Anesthesiology and intensive care	35	5.5
Cardiology	29	4.6
Dermatology	29	4.6
Clinical pathology, pharmacology, medical parasitology and microbiology	28	4.4
Rheumatology and rehabilitation	23	3.6
Family medicine	15	2.4
Hepatology and gastroenterology	15	2.4
Pneumology	14	2.2
Nephrology	13	2.0
Medical oncology & nuclear medicine	10	1.6
Clinical nutrition	5	0.8
Audiology	4	0.6
Phonetics	4	0.6
Public health and infection control	4	0.6
Hematology	4	0.6
Tropical medicine	3	0.5
Other specialties^a^	3	0.5
Surgery^e^	325	33.9
Gynecology and obstetrics	73	22.4
General surgery	56	17.2
Orthopedics	47	14.4
Dentistry and oral surgery	42	12.9
Ophthalmology	37	11.4
Otorhinolaryngology	21	6.5
Urosurgery	16	4.9
Brain and neurosurgery	9	2.7
Plastic and reconstructive burn surgery	8	2.4
Vascular surgery	7	2.1
Pediatric surgery	4	1.2
Cardiothoracic surgery	3	0.9
Maxillofacial surgery	2	0.6
Workplace^f^		
One job; governmental public health sector	261	27.2
One job; private health sector	90	9.4
Dual government-private job	608	63.4
Private health sector^f^ (*n* = 698)		
Own a private clinic	261	37.4
Share in a private clinic^d^	157	22.5
Work at an outpatient clinic at private hospital	412	59.0
Governmental public health sector^f^ (*n* = 869)		
UTH	150	17.1
MoHP	644	73.6
HIO	56	6.4
Dual jobs^c^	19	2.1

Abbreviations: *UTH* University Teaching Hospitals, *MoHP* Ministry of Health and Population, *HIO* Health Insurance Organization

^a^Endocrinology, clinical toxicology

^b^For each subspecialty, the percentage was calculated among total number of internists (*n* = 634)

^c^Some professionals had dual MOHP-HIO jobs (*n* = 13), University-MoHP jobs (*n* = 5), or University-HIO jobs (*n* = 1)

^d^Work with colleagues of same or other specialties in private clinic and share in management

^e^For each subspecialty in surgery, the percentage was calculated among total number of surgeons (*n* = 325)

^f^Categories are not mutually exclusive

### Satisfaction with job and salary at governmental and private work

**Fig. 1:**
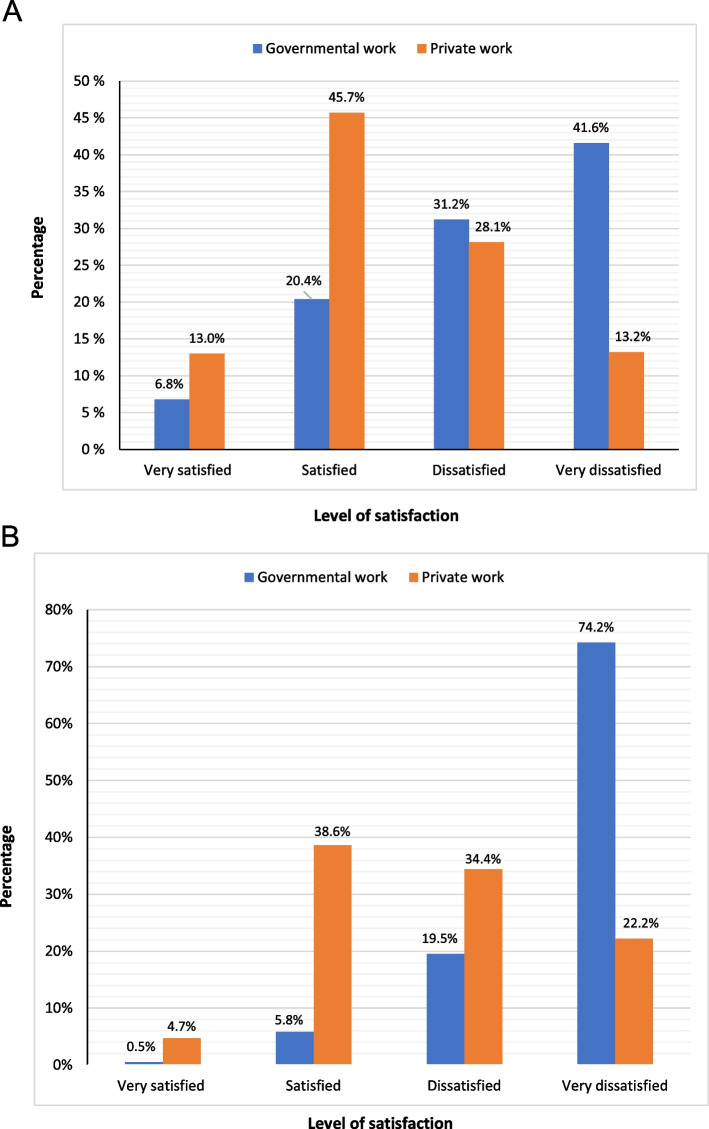
**A** Level of satisfaction with job at the governmental (*n* = 827)^a ^and private (*n* = 698) health sectors among medical professionals in Egypt, 2021. **B** Level of satisfaction with salary at the governmental (*n* = 827)^a^ and private (*n* = 698) health sectors among medical professionals in Egypt, 2021. ^a^Among professionals at governmental sector (*n* = 869), 827 reported level of satisfaction, whereas 42 did not

More than half of professionals at the private sector reported being satisfied or very satisfied with their private job (58.7%), whereas less than half were satisfied or very satisfied with their private salary (43.3%). On the contrary, the majority of professionals at the governmental sector were dissatisfied or very dissatisfied with the government job (72.8%) and salary (93.7%) (Fig. 1 A, B).

Dissatisfaction with the government salary was independently predicted by working at the MoHP (OR = 5.54, 95%CI = 2.39, 12.8; *p* < 0.001). The multivariate logistic regression model correctly classified 93.7% of professionals for dissatisfaction with government salary (Tables 2 and 3). As for private work, significant association was found between job satisfaction and working with colleagues of the same or different specialties (OR = 1.56, 95%CI = 1.07, 2.26; *p* = 0.01).

**Table 2:** Factors associated with dissatisfaction with job and salary at the governmental public health sector (*n* = 827) (Professionals at governmental sector who reported level of satisfaction)

Factors	Satisfied^a^ (*n* = 52)		Dissatisfied^b^ (*n* = 775)		OR (95%CI)	*P* value
	(No.)	(%)	(No.)	(%)		
Dissatisfaction with job						
Gender						
Male	128	26.3	359	73.7	1.12 (0.82, 1.52)	0.47
Female^d^	97	28.5	243	71.5		
Age (years)						
≥ 40	30	23.6	97	76.4	1.24 (0.80, 1.94)	0.32
< 40^d^	195	27.9	505	72.1		
Specialty						
Internal medicine^d^	146	26.6	403	73.4	1.09 (0.79, 1.51)	0.57
Surgery^d^	79	28.4	199	71.6		
Workplace						
University HC system^d^	52	34.7	98	65.3	ref	-
MOHP	157	25.1	469	74.9	1.58 (1.08, 2.32)	0.01^*^
HIO	16	31.4	35	68.6	1.16 (0.58, 2.29)	0.66
Dissatisfaction with salary						
Gender						
Male	23	4.7	465	95.3	1.89 (1.07,3.33)	0.02^*^
Female^d^	29	8.6	310	91.4		
Age (years)						
≥ 40	7	5.6	119	94.4	1.16 (0.51, 2.64)	0.71
< 40^d^	45	6.4	656	93.6		
Specialty						
Surgery	12	4.3	266	95.7	1.74 (0.89, 3.37)	0.09
Internal medicine^c,d^	40	7.3	509	92.7		
Workplace						
UTH	19	12.8	130	87.2	1.43 (0.60, 3.41)	0.41
MOHP	24	3.8	602	96.2	5.25 (2.29, 11.9)	< 0.001^***^
HIO^d^	9	17.3	43	82.7	ref	-

Abbreviations: *HC* health care, *UTH* University Teaching Hospital, *MoHP* Ministry of Health and Population, *HIO* Health Insurance Organization, *OR* odds ratio, *CI* confidence interval

^a^Satisfied or very satisfied

^b^Dissatisfied or very dissatisfied

^c^Internists, general practitioners, and radiologists

^d^Reference category

^*^P < 0.05

^***^P < 0.001

**Table 3:** Multivariate logistic regression analysis of independent predictors of dissatisfaction with government salary (*n* = 827)^b^

Independent predictors	Coefficient	Adjusted OR^a^	95% CI	*P*-value
Male gender	0.564	1.757	(0.94, 3.25)	0.073
Age > = 40 years	0.244	1.276	(0.55, 2.96)	0.570
Surgical specialty	0.300	1.350	(0.65, 2.79)	0.420
Working at UTH	0.434	1.543	(0.63, 3.75)	0.339
Working at MoHP	1.713	5.548	(2.39, 12.8)	< 0.001^***^
Model χ^2^ = 29.23 (*p* < 0.001); Nagelkerke’s R^2^ = 0.1; Hosmer & Lemeshow χ^2^ = 4.551 (*p* = 0.603)				

Abbreviations: *MoHP*, Ministry of Health and Population; *UTH*, University Teaching Hospital; *OR*, odds ratio; *CI*, confidence interval

^a^OR adjusted for all variables in the above table

^b^medical professionals at governmental sector who reported level of satisfaction with salary

^***^P < 0.001

### Pros and cons of working at governmental and private sectors

Regarding the governmental sector, the most reported pros (addressed by 640 professionals) were providing health services to a large number of deprived populations (18.28%); opportunity to develop skills (10.5%), cooperation of colleagues at work (8.1%); and stable job with guaranteed slowly rising salary, insurance, and pension (5.1%). On the contrary, 659 professionals reported the following cons: jobs are underpaid with a lack of appreciation and recognition by the government (37.8%), insufficient resources (28.37%), corruption of the healthcare system, and bad management and abusive supervision (24.6%).

Regarding the private sector, the most reported pros (addressed by 534 professionals) were well-paid jobs relative to governmental jobs (45.9%), availability of resources (9.5%), and appropriate workplace environment (7.7%). On the other hand, 526 professionals reported the following cons: jobs are underpaid (28.3%), exhausting work both physically and psychologically (19.8%), and capitalism in management that interferes with principles of the profession (13.8%).

### Potential solutions to improve medical practice in Egypt

**Fig. 2:**
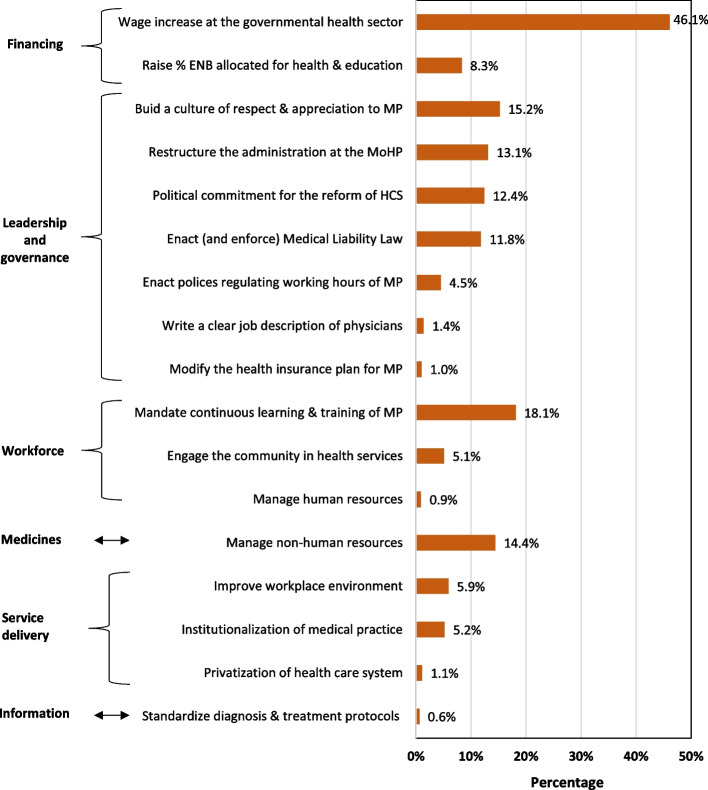
Potential solutions^*^ to improve medical practice in Egypt (*n* = 959). Abbreviations: MoHP, Ministry of Health and Population; ENB, Egypt national budget; HCS, health care system; MP, medical professionals. ^*^More than one solution could be suggested by the participant

Professionals’ proposed solutions were organized according to the World Health Organization health systems framework into six building blocks (24) (Fig. 2).

1. Leadership and governance

It would be important to build a culture of respect and appreciation for medical professionals by the higher authorities and public (15.2%), restructure administration at MoHP (13.1%), ensure political commitment for the healthcare system reform (12.4%), and enact and enforce Medical Liability Law with continuous supervision (11.8%)

2) Financing

Wage increase for physicians at the governmental health sector (to reduce their need to join the private sector) was the most frequently reported solution (46.1%). In addition, rising the percentage of Egypt National Budget allocated for health and education sectors (8.3%)

3) Workforce

It was highly suggested to mandate continuous training of physicians (18.1%), engage the community in health services (5.1%), and manage human resources (0.9%)

4) Medicines

Management of non-human resources (medical equipment, pharmaceuticals, and materials) was recommended by 14.4% of professionals

5) Information

Professionals recommended the dissemination of standardized diagnosis and treatment protocols at all healthcare facilities (0.6%)

6) Service delivery

It was recommended to improve the workplace environment (5.9%) and develop the poorly functioning governmental sector (with respect to quality, access, safety, and coverage) and to gradually eliminate the private sector and stop the segmentation of the healthcare system “institutionalization” (5.2%).

### Telemedicine perception and practice

**Fig. 3:**
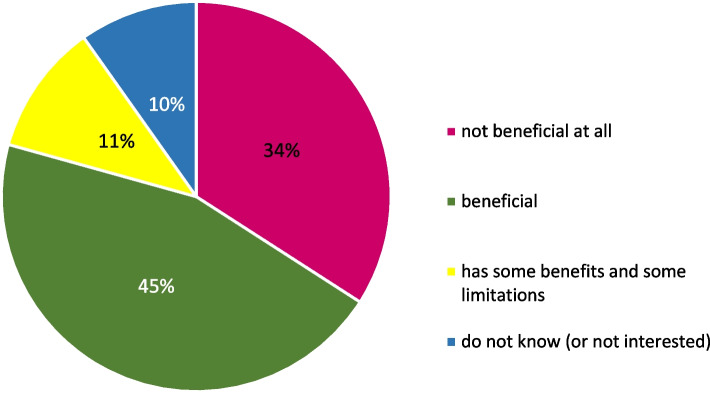
Perception of the benefits of telemedicine by the medical professionals in Egypt, 2021 (*n* = 959)

During the COVID-19 pandemic, 90.6% of professionals practiced telemedicine. Regarding perception of telemedicine, 9.8% of professionals were not interested (or did not know about it) and 34.1% found it not beneficial at all. On the contrary, 45.2% found it beneficial or has some benefits and some limitations (10.9%) (Fig. 3). A significant association was found between the perception of the benefits of telemedicine and gender and medical specialty. The odds of perception of benefits of telemedicine were two times higher in women than in men (OR = 2.00, 95%CI = 1.49, 2.67; *p* < 0.001) and 1.39 times higher in internists than in surgeons (OR = 1.39, 95%CI = 1.05,1.86; *p* = 0.02).

### Benefits and disadvantages of telemedicine

**Fig. 4:**
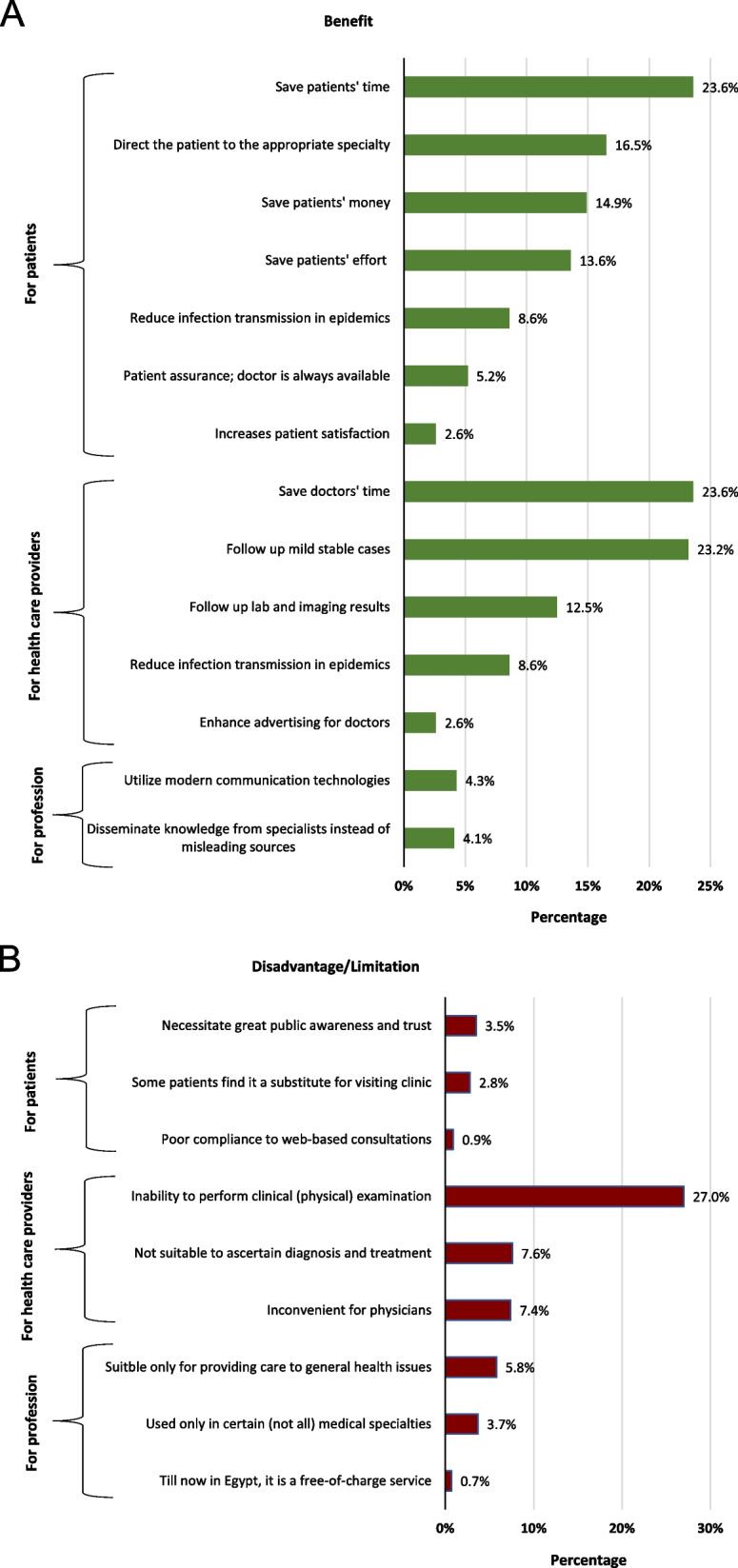
**A** Benefits^*^ of telemedicine, as perceived by medical professionals in Egypt, 2021 (*n* = 548^^^). ^^^Professionals who found telemedicine beneficial or has some benefits; ^*^More than one benefit could be addressed by the participant. **B** Disadvantages^#^ of telemedicine, as perceived by medical professionals in Egypt, 2021 (*n* = 432^&^). ^&^Professionals who found telemedicine challenging or has some limitations; ^#^More than one disadvantage could be addressed by the participant

As stated by professionals who found telemedicine beneficial or has some benefits (*n* = 548), benefits were grouped into three categories (Fig. 4).

1. Benefits for the patients

Telemedicine saves patients’ time (23.6%), money (14.9%), and effort (13.6%) especially when patient transfer is difficult. In addition, it reduces infection transmission during epidemics (8.6%).

2) Benefits for the health care providers

Telemedicine saves doctors’ time (23.6%), allows follow up on mild stable cases with known medical history and who have previously been clinically examined (23.2%), and follow up of laboratory and imaging results (12.5%).

3) Benefits for the profession

Telemedicine utilizes modern communication technology, especially the interactive ones such as “video conferencing” (4.3%) and helps to disseminates general medical advice and health education (2.8%), so people gain knowledge from specialists instead of seeking misleading sources (4.1%).

On the other hand, potential limitations indicated by professionals who found telemedicine challenging or has some limitations (*n* = 432) were organized into three categories (Fig. 4).

1. Disadvantages/limitations for the patients

Telemedicine necessitates greater public awareness and trust; people should be able to effectively describe their symptoms, complains, and needs (3.5%). In addition, some patients find it a substitute for visiting the clinic, and others might abuse it (2.8%).

2) Disadvantages/limitations for the health care providers

The most frequently stated disadvantage was the inability to perform clinical examination; web-based consultations do not replace face-to-face physician-patient communication (27.0%).

3) Disadvantages/limitations for the profession

Telemedicine is suitable only for general medical issues (5.8%) and in certain (not all) medical specialties (3.7%).

Fifty-four professionals stated that telemedicine in Egypt needs to be practiced within a legal framework that ensures licensing of medical personnel and authentication of users, by means of applications that regulate time and payment issues, and integrates services provided by physicians, laboratories, and imaging centers.

## Discussion

The present study revealed low to moderate job satisfaction; 27.2% at the governmental and 58.7% at the private sector. Underpayment was the most reported disadvantage in both sectors. Wage increase, management of non-human resources, and continuous professional training were the most proposed solutions. During the COVID-19 pandemic, the majority of professionals practiced telemedicine (90.7%) with a moderate level of perception of its benefits (56%).

### Job satisfaction

The percentage of professionals who reported satisfaction with their government job (27.2%) was lower than that reported in an Egyptian study in 2008, where 38.7% had expressed overall job satisfaction (21). Though this could indicate a downward trend when it comes to job satisfaction, another explanation related to the timing of the study could be considered. The present study was conducted during the COVID-19 pandemic. Job satisfaction was found to be negatively associated with the fear of COVID-19 among medical professionals in Abd Ellatif et al. study (2021) (28). In the present study, relatively more professionals have expressed satisfaction at the private sector compared with the governmental sector. Within the governmental sector, dissatisfaction was significantly associated with working at MoHP compared with HIO. Such a comparison, as far as we know, has not been evaluated by a study in Egypt yet.

### Proposed solutions to improve medical practice

In the present study, the most reported disadvantage of the healthcare system and most proposed solutions were related to healthcare financing. This finding denotes a requirement for analysis of the structure and dynamics of healthcare financing in Egypt. According to Fasseeh et al. systematic review (2022), in Egypt, governmental health expenditure (GHE) represented approximately one third of the total health expenditure (THE). In the last 12 years, although THE as an absolute value was increasing, ranging from 139 to 393 billion EGP, THE as a percent of gross domestic product (GDP) was almost stagnant or even declining, with a median of 5.5%. Recently reported GHE (as a percentage of GDP) was 3% (29). However, according to the constitutional obligation in 2014, GHE should gradually rise to match the global levels of THE as a percentage of GDP, which is around 10% (30).

Moreover, the primary healthcare financing source in Egypt is out-of-pocket expenditure, representing more than 60% of THE, followed by government spending through the Ministry of Finance (37% of THE) (29). Implementation of Universal Health Insurance in the next period is expected to drive the Egyptian healthcare market to a more governmentally funded system, thus reducing the gigantic out-of-pocket expenditure (29, 31, 32).

In the present study, the second most proposed solution to improve medical practice was providing mandatory continuous training to medical professionals (18.1%). This supports findings in Schumann et al. study (2019), where searching for training opportunities was found to be a driving factor for migration of Egyptian physicians (20).

### Telemedicine in Egypt

During the COVID-19 pandemic, 90.6% of medical professionals in the current study practiced telemedicine with moderate level of perception of its benefits (56%). This perception level is similar to that reported in Iran (33); however, a study conducted in Saudi Arabia reported a substantially higher favorable perception level (90%) (34). Professionals’ knowledge about telemedicine and their technology-related skills might have influenced their perception; however, those factors were not evaluated in the current study.

Benefits of telemedicine reported in the current study were similar to those reported in earlier studies (34–37). On the contrary, the most reported disadvantage of telemedicine in the current study was the breakdown of the physician–patient relationship and the inability to perform physical examination (27%). This finding is in agreement with previous findings (35, 36), where it has been suggested that the risk of breakdown in the relationship might be related to factors such as lack of formal training in using telemedical equipment, physical, or mental factors such as suffering from a reduced vision or difficulty of hearing, depersonalization where teleconsultation might be experienced as watching TV not as being real communication, lack of confidence of patients and professionals, and inability to perform entire physical examination over a video link (35).

Medical professionals in the current study emphasized that telemedicine needs to be legalized in Egypt. An overview of the legislative and policy trend in relation to regulating digital matters suggests that Egypt is adopting a reactive approach that responds to issues with a sometimes-considerable delay (7). For digitalization to achieve its prospects, Egypt needs a nationwide digital ecosystem including universally affordable high-speed Internet, a human capital asset with digital skills, digital platforms, interoperable digital payment services, digital identities, and a legal and regulatory environment (38).

### Limitations of the study

Though the current study was able to widely represent working medical professionals in Egypt, the great diversity in work circumstances at the governmental and private sectors hindered a full in-depth comparison to identify determinants of job satisfaction. In addition, an evaluation of the knowledge about telemedicine and technology-related skills would have enabled studying their independent effect on the perception of telemedicine.

## Conclusions

During the COVID-19 pandemic in Egypt, medical professionals reported low to moderate job satisfaction and a moderate level of perception of the benefits of telemedicine. The study highlighted the need to analyze healthcare financing in Egypt and provide continuous training to medical professionals. In addition, future research is required to identify factors related to satisfaction with digital health in general and telemedicine in particular.

## Data Availability

Data and materials are available on reasonable request.

## Abbreviations

GDP: Gross Domestic Product
GHE: Governmental Health Expenditure
HIO: Health Insurance Organization
MoHP: Ministry of Health and Population
THE: Total Health Expenditure
